# Single Charge Current in a Normal Mesoscopic Region Attached to Superconductor Leads via a Coupled Poisson Nonequilibrium Green Function Formalism

**DOI:** 10.1155/2014/721671

**Published:** 2014-04-09

**Authors:** David Verrilli, F. P. Marin, Rafael Rangel

**Affiliations:** ^1^Laboratorio de Física Teórica de Sólidos (LFTS), Centro de Física Teórica y Computacional (CEFITEC), Facultad de Ciencias, Universidad Central de Venezuela, A.P. 47586, Caracas 1041-A, Venezuela; ^2^Departamento de Física, Universidad Simón Bolívar, A.P. 89000, Caracas 1080-A, Venezuela

## Abstract

We study the *I*-*V* characteristic of mesoscopic systems or quantum dot (QD) attached to a pair of superconducting leads. Interaction effects in the QD are considered through the charging energy of the QD; that is, the treatment of current transport under a voltage bias is performed within a coupled Poisson nonequilibrium Green function (PNEGF) formalism. We derive the expression for the current in full generality but consider only the regime where transport occurs only via a single particle current. We show for this case and for various charging energies values *U*_0_ and associated capacitances of the QD the effect on the *I*-*V* characteristic. Also the influence of the coupling constants on the *I*-*V* characteristic is investigated. Our approach puts forward a novel interpretation of experiments in the strong Coulomb regime.

## 1. Introduction

The overall shape of the *I*-*V* characteristic of a variety of systems (metals, semiconductors, and molecular conductors) in the nanometer scale sandwiched between metallic or superconductors leads has been recently a matter of study (see [1, 2] and references therein). In these systems, the energy level discreteness is quite important since level spacing is comparable with other energy scales [3, 4]. Indeed, the coupling with the bath modifies drastically the properties of an otherwise uncoupled nanometer system in a sharp contrast with similar nonequilibrium macroscopic systems [5–13]. They constitute hybrid systems. Theoretical studies [14–20] as well as experimental measurements have been done by many research groups [1–3] on such systems mostly at low enough temperature with negligible thermal and non-equilibrium fluctuations.

**Figure 1:**
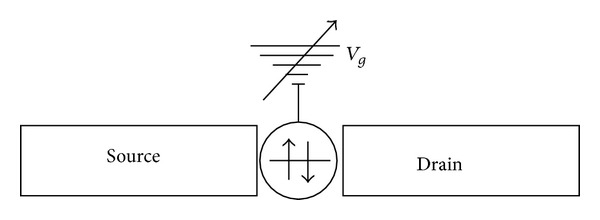
Set-up: single level quantum dot connected with two superconducting leads via coupling constants Γ_*s*_.

**Figure 2:**
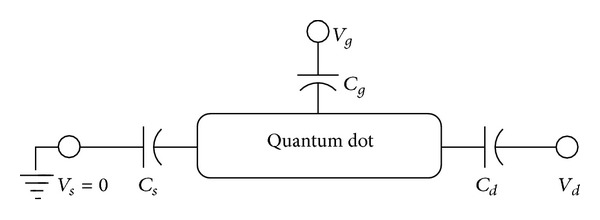
Equivalent capacitive circuit with coupling capacitances *C*_*s*_, *C*_*g*_, and *C*_*d*_, corresponding to the capacitances in the source, gate and drain, respectively.

All the systems mentioned above underlay universal common features with the hybrid superconductor quantum dot devices we want to address in this work [2, 4, 21, 22]: (i) broadened energy levels of the quantum dot due to hybridization with the leads; (ii) spatial potential profile. (iii) a charging energy *U*_0_ due to the potential profile. An insight behind these issues has been highlighted recently [23, 24] for molecular dots. The device we study in this work is shown in Figure 1. It constitutes a spin degenerated quantum dot level, which is coupled to a pair of biased superconductors contacts or leads (source and drain). When a source-drain voltage *V*_*d*_ is applied, an electric current flows between the leads and across the quantum dot. The biasing defines a non-equilibrium steady state situation. Such situation is coming from the frustration to establish simultaneously an equilibrium configuration with both leads under a given bias. In addition, a gate voltage *V*_*g*_ sets the quantum dot spectrum. However, the charge energy can modify it whenever the density of states is significant. In response to the applied voltages, an actual potential develops inside the dot; that is, an effective electrostatic profile potential inside the mesoscopic region exists in such a way, that it couples to both the electronic non-equilibrium state population and the non-equilibrium electric current. That approach, as introduced by Datta [4], links the electrostatic profile to the electronic population of the quantum dot [4, 25] via the non-equilibrium Keldysh formalism (NEGF) [26, 27]. The whole system is modeled by coupling capacitances which represents the drain, source, and gate contributions to the self-consistent electrostatic problem. Incoming electrons have to overcome an energy barrier (Coulomb blockade). On the other hand, gate or source-drain voltage can lower or increase this energy barrier. These source, drain, and gate electrodes capacitances (see Figure 2) constitute a simple capacitive model (in experiments [2, 28], these capacitances are measured) from which *U*_*ℒ*_, the Laplacian part of the potential, can be obtained. In addition, the charge in dot can be expressed as the sum of the charges in the coupling capacitances. It yields the Poisson contribution *U*_*P*_ to the total potential *U*, as a function of the dot population. In other words, we solve the self-consistency (SC) of the total electrostatic potential *U* = *U*_*ℒ*_ + *U*_*P*_ together with the dot population. After that, the electric current is evaluated.

Previous to the self-consistent program, the non-equilibrium current through the dot and electronic occupation in the dot are worked out. We emphasize that the calculation is carried out in a general framework. However, we confine our attention to the single particle current contribution. We adapt the SC to two different approximation regimes. In Section 4, the equivalent capacitive circuit (Figure 2) is introduced; the spatial potential profile *U* is calculated within the capacitive model. The SC scheme is applied to two cases [29, 30]. First, the so-called restricted case, where the gap is the bigger energy scale and the coupling QD-Leads is of the order of the charging energy (Δ ≫ Γ_*L*,*R*_≃*U*). In this case, quantitative results are expected to be accurate. We also make calculations for the so-called unrestricted case, where the charging energy is the dominant energy scale Δ≃*U* ≫ Γ_*L*,*R*_. In this case the results are quantitatively less accurate. The experiments of Ralph et al. [28] were done in this regime. Their *I*-*V* characteristic shows that the spacing of the energy levels is subjected to strong fluctuations. According to our model, the fluctuations are due to complex multilevel charging effects. Our hybrid S/QD/S system has been studied in previous theoretical works [16–19]. However, to our knowledge, the coupled SC scheme which describes charging effects has not been considered so far. This is an important step; then, gauge invariant independence of the results as well independence of the zero reference voltage is fulfilled [31, 32]. Our model uses experimental values of the equivalent capacitances [4]. To this respect, pioneering work is done by Meir et al. [33, 34] for N/QD/N systems, considering the interatomic Coulomb term *Un*_↑_ *n*_↓_ as a measure of the charging energy *e* ^2^/*C*. Their purpose was to find the main object of the non-equilibrium formalism, namely, the QD Green-Keldysh function, in which the influence of the leads on the QD is taken into account. Due to the presence of the Coulomb term, its equation of motion generates a two-particle Green-Keldysh function. By ignoring correlations with the leads, the equation of motion for the QD Green function closes after truncation of higher order equations of motion. This solution (their Equation (8)) has two resonances, one at the energy level weighted by the probability that the other spin degenerate level (raised by *U*) is vacant and another one at the energy level raised by *U* weighted by the probability that the level is occupied. It is correct for temperatures higher than the Kondo temperature and is exact in the noninteracting limit (*U* = 0) and the isolated limit. Analogously, for S/QD/S hybrid systems Kang [16] has obtained an expression for the current through the QD (his Equation (8)), which is evaluated in the *U* → *∞* limit (his Equation (13)). The QD Green function from the very beginning does not contain off-diagonal terms that involve superconducting pairing, which excludes the possibility of Andreev reflection processes. The presence in the equation for the current (his Equation (14)) of terms proportional to (1 − 〈*n*_−*σ*_〉) affects the contribution to the current of the considered level. In order to complete the outlined program one has to calculate 〈*n*_−*σ*_〉 self consistently which is not carried out. Instead, Kang calculate the current (his Equation (8)) where the spectral function is calculated in the limit of zero coupling with the leads via a model taken from literature (his Reference [18]) and without taking into account the dependence of the contribution of one level to the current on the occupancy of the other. The point of view which neglects the unavoidable influence of the bath (the leads) on the small system (the QD) is accomplished by factorizing the density matrix (*ρ*(*t*) = *ρ*_QD_⊗*ρ*_Baths_) and integrating out the leads degrees of freedom which simplify the Liouville-von Neumann equation (Equation (3.140) in [35]). This program is carried out by Kosov et al. for S/QD/S system [36]. In this way, a Markovian master equation is obtained and an expression for the current is calculated. In their Figure 2, they show the *I*-*V* characteristic of a nondegenerated QD for a given set of parameters. In this case, the Cooper pair density in the QD is zero [37]. For the sake of comparison, we restrict our calculations to this case. A similar but not identical approach was done by Pfaller et al. [38]. Also, the approach of both Kosov et al. and Pfaller et al. misses the energy levels broadening as discussed in the introduction. This lack of broadening is a general deficit of quantum Markov approach [39]. In particular, Pfaller et al. [38] introduce a phenomenological broadening while our approach derives it from first principles. In fact, within the Keldysh formalism, this broadening appears naturally (see (58) below). Levy Yeyati et al. [17] writes an expression for the current (his Equation (2) and Figure 2). They use that expression to explain the experimental results of Ralph et al. [28]. Their calculation was done in the *U* → *∞* limit. In addition, they include charging effects, although they do not say explicitly in which way these effects are included. In this respect, one has to realize that *U* has important contributions to the QD mesoscopic charging effect. In *t* → −*∞*, the leads and the QD maintain independent thermal equilibrium, that is, are uncoupled systems. When they become coupled, the Keldysh formalism yields the general behavior of the system. After a long enough time, this particular system reaches a steady state.

Our point of view is taken from the fact that the charging of the QD is the origin of the Coulomb repulsion between two electron is occupying a two-fold degenerate level. Therefore, we study the behavior of a noninteracting QD at *t* → −*∞* where exact expressions are found. In this way, we obtain a formally similar expression (see (61) below) for the current as Equation (12) in the work of Meir and Wingreen [33]. Later on, Coulomb repulsion is introduced via a self-consistent field (SCF) that depends dynamically on the applied bias (*H*_QD_ + *U*_SCF_) and, in consequence, on the actual number of electrons in the QD. This approach constitutes the coupled Poisson NEGF formalism that has been discussed in the context of molecular conductors by Datta et al. [4, 24]. We use a capacitive model in Section 4 to calculate *U*_SCF_ and as discussed above, a numerical procedure is used to evaluate the current. Our approach has the known disadvantage of ignoring correlations in the QD (as pointed out in [39]). In that sense, there is a proposal by Datta (Equation (3.4.9) in [4]) that improves the SCF method and permits more accurate quantitative results. In Section 4, we apply this improvement for the case when the Coulomb charging is greater than the value of the coupling constants. We discuss possible improvements of our approach in Section 6.

## 2. Single Level QD-Model: Derivation of Nonequilibrium Currents

In macroscopic systems, the task of deriving transport equations or generalized Ginzburg-Landau equations relies on quasiclassical Green functions [7]. In addition, recently non-equilibrium transport in dirty Aluminium quasi-one-dimensional nanowires coupled with normal reservoirs [11] was studied experimentally and theoretically with quasi-classical Green functions [13]. As we want to include the possibility of particle interference effects, we do no resort to such objects. This point of view has been discussed in [14]. Instead, we use the equation of motion method (EOM) technique of Keldysh formalism for generating non-equilibrium states (see [8–10, 26]). We consider a spin degenerated single orbital as a quantum dot connected to superconductors leads. The Hamiltonian which describes this system is a generalized Anderson model [40]. It reads

(1) $$\begin{array}{c} H=H_{S}+H_{\text{QD}}+H_{T}, \end{array}$$

where *H*_*S*_, *H*_QD_, and *H*_*T*_ stand for the superconducting leads, the dot, and the tunneling term, respectively. Also *H*_*S*_ = ∑_*η*_ *H*_*η*_ = *H*_*L*_ + *H*_*R*_, where *H*_*L*_ and *H*_*R*_ are the left and right lead Hamiltonians, respectively. They are given, within the BCS model [41], by

(2) $$\begin{array}{c} H_{S}=\underset{\eta \vec{k}\sigma }{\sum }\Psi_{\eta \vec{k}\sigma }^{†}H_{\eta \vec{k}}^{0}\Psi_{\eta \vec{k}\sigma }, \end{array}$$

with

(3) $$\begin{array}{c} H_{\eta \vec{k}}^{0}=\left(\begin{array}{cc} \varepsilon_{\eta \vec{k}} & \Delta_{\eta \vec{k}}\\ \Delta_{\eta \vec{k}}^{*} & -\varepsilon_{\eta \vec{k}} \end{array}\right), \end{array}$$

where $\varepsilon_{\eta \vec{k}}$ is the conduction electron energy and $\Delta_{\eta \vec{k}}$ is the superconductor gap of the lead *η* = *L*, *R*. $\Psi_{\eta \vec{k}\sigma }^{†}$ and $\Psi_{\eta \vec{k}\sigma }$ are the Nambu spinors:

(4) $$\begin{array}{c} \Psi_{\eta \vec{k}\sigma }^{†}=\left(\begin{array}{cc} a_{\eta \vec{k}\sigma }^{†} & a_{\eta ,-\vec{k},-\sigma } \end{array}\right),\;\;\Psi_{\eta \vec{k}\sigma }=\left(\begin{array}{c} a_{\eta \vec{k}\sigma }\\ a_{\eta ,-\vec{k},-\sigma }^{†} \end{array}\right). \end{array}$$

Here $a_{\eta \vec{k}\sigma }^{†}\left(a_{\eta \vec{k}\sigma }\right)$ denotes the creation (annihilation) operator for a conduction electron with wave vector $\vec{k}$ and spin *σ* in the *η* = *L*, *R* superconductor lead.

*H*_QD_ is the hamiltonian for the single-level quantum dot of energy *E*_0_:

(5) $$\begin{array}{c} H_{\text{QD}}=\underset{\sigma }{\sum }\phi_{\sigma }^{†}H^{\text{QD}}\phi_{\sigma }, \end{array}$$

with

(6) $$\begin{array}{c} H^{\text{QD}}=\left(\begin{array}{cc} E_{0} & 0\\ 0 & -E_{0} \end{array}\right). \end{array}$$

The model QD does not contain the Hubbard Coulomb repulsion interaction term. As explained in the introduction, Coulomb repulsion is modeled by means of the inclusion of capacitances, which are taken independent of the charge in the QD. The model also ignores possible superconducting correlations in the QD. For sufficiently small QDs, the discreteness of the single energy levels suppresses these correlations [37]. The position of the energy level will be treated first as fixed by the gate potential with respect to the left lead, while the effect of the applied voltage is taken into account by the coupled Poisson scheme. The tunneling hamiltonian *H*_*T*_ is given by

(7) $$\begin{array}{c} H_{T}=\underset{\eta \vec{k}\sigma }{\sum }\Psi_{\eta \vec{k}\sigma }^{†}H_{\eta \vec{k}}^{I}\phi_{\sigma }, \end{array}$$

with

(8) $$\begin{array}{c} H_{\eta \vec{k}}^{I}=\left(\begin{array}{cc} V_{\eta \vec{k}} & 0\\ 0 & -V_{\eta \vec{k}} \end{array}\right). \end{array}$$

*H*_*T*_ connects the dot to the biased superconducting leads and it allows the electric charge flow. $V_{\eta \vec{k}}$ is the hybridization matrix element between a conduction electron in the *η* = *L*, *R* superconductor lead and a localized electron on the dot with energy *E*_0_. *ϕ*_*σ*_^†^ and *ϕ*_*σ*_ are the dot spinors:

(9) $$\begin{array}{c} \phi_{\sigma }^{†}=\left(\begin{array}{cc} d_{\sigma }^{†} & d_{-\sigma } \end{array}\right),\;\;\phi_{\sigma }=\left(\begin{array}{c} d_{\sigma }\\ d_{-\sigma }^{†} \end{array}\right); \end{array}$$

here, *d*_*σ*_^†^(*d*_*σ*_) is the creation (annihilation) operator for an electron on the dot.

The flow of electric charge from the terminal *η* is given by

(10) $$\begin{array}{c} I_{\eta }\left(t\right)=\left(-e\right)\left[-\frac{d\langle N_{\eta }\left(t\right)\rangle }{dt}\right]=\frac{\text{i}e}{\hbar }\langle \left[H_{T}\left(t\right),N_{\eta }\left(t\right)\right]\rangle , \end{array}$$

where −*e* is the electron charge. 〈⋯〉 is the thermodynamical average over the biased *L* and *R* leads at the temperature *T*, taken at time *t*_0_ → −*∞*, as indicated in the Keldysh contour in Appendix A:

(11) $$\begin{array}{c} \langle \cdots \rangle \equiv \mathrm{Tr}\left(\rho \left(t_{0}\right)\cdots \right),\;\;\rho \left(t_{0}\right)\equiv \frac{e^{-\beta (H-\mu N)}}{\mathrm{Tr}\left(e^{-\beta (H-\mu N)}\right)}, \end{array}$$

and $N_{\eta }=a_{\eta \vec{k}\sigma }^{†}a_{\eta \vec{k}\sigma }$ is the “number of particles” operator. Book-keeping calculation using (10) leads to

(12) $$\begin{array}{c} I_{\eta }\left(t\right)=\frac{2e}{\hbar }V_{\eta }ℜ\underset{\vec{k}\sigma }{\sum }{\text{F}}_{\eta \vec{k}\sigma }^{<}\left(t,t\right). \end{array}$$

${\text{F}}_{\eta \vec{k}\sigma }^{<}\left(t,t^{\prime }\right)=\text{i}\langle d_{\sigma }^{†}\left(t^{\prime }\right)a_{\eta \vec{k}\sigma }\left(t\right)\rangle$ is the lesser Keldysh Green function,

(13) Fηk→σ(t,t′) ≡−i〈TKaηk→σ(t)dσ†(t′)〉 ≡−iΘ(t,t′)〈aηk→σ(t)dσ†(t′)〉+iΘ(t′,t)  ×〈dσ†(t′)aηk→σ(t)〉 ≡Θ(t,t′)Fηk→σ>(t,t′)+Θ(t′,t)Fηk→σ<(t,t′),

and T_K_ is the time-ordering operator, the action of which is to rearrange product of operators, such that operator with later times, on the Keldysh contour are placed to the left of the product. Hereafter, for simplicity, we replace $V_{\eta \vec{k}}$ by an average *V*_*η*_ at the Fermi surfaces ($V_{\eta \vec{k}}\equiv \sqrt{{\langle |V_{\eta \vec{k}}{|}^{2}\rangle }_{\text{FS}}}$) of the leads *L* and *R*. Using the scheme given in Appendix A for the rate of change of (13), we proceed to obtain the equation of motion:

(14) i∂Fηk→σ(t,t′)∂t =δ(t,t′)〈{aηk→σ(t),dσ†(t)}〉−i〈TK[aηk→σ(t),H]dσ†(t′)〉,

which leads to

(15) $$\begin{array}{c} \left(\text{i}\frac{\partial }{\partial t}-\epsilon_{\eta \vec{k}}\right){\text{F}}_{\eta \vec{k}\sigma }\left(t,t^{\prime }\right)=-\sigma \Delta_{\eta }{ℱ}_{\eta \vec{k}\sigma }\left(t,t^{\prime }\right)+V_{\eta }{\text{G}}_{\sigma }\left(t,t^{\prime }\right), \end{array}$$

where

(16) $$\begin{array}{c} {ℱ}_{\eta \vec{k}\sigma }\left(t,t^{\prime }\right)=-\text{i}\langle {\text{T}}_{\text{K}}a_{\eta \vec{k},-\sigma }^{†}\left(t\right)d_{\sigma }^{†}\left(t^{\prime }\right)\rangle , \end{array}$$

(17) $$\begin{array}{c} {\text{G}}_{\sigma }\left(t,t^{\prime }\right)=-\text{i}\langle {\text{T}}_{\text{K}}d_{\sigma }\left(t\right)d_{\sigma }^{†}\left(t^{\prime }\right)\rangle . \end{array}$$

Note that G_*σ*_(*t*, *t*′) is the QD single-particle Green function. Similarly, ${ℱ}_{\eta \vec{k}\sigma }\left(t,t^{\prime }\right)$ satisfies the equation of motion:

(18) $$\begin{array}{c} \left(\text{i}\frac{\partial }{\partial t}+\epsilon_{\eta \vec{k}}\right){ℱ}_{\eta \vec{k}\sigma }\left(t,t^{\prime }\right)=-\sigma \Delta_{\eta }{\text{F}}_{\eta \vec{k}\sigma }\left(t,t^{\prime }\right)-V_{\eta }{𝒢}_{\sigma }\left(t,t^{\prime }\right), \end{array}$$

where

(19) $$\begin{array}{c} {𝒢}_{\sigma }\left(t,t^{\prime }\right)=-\text{i}\langle {\text{T}}_{\text{K}}d_{-\sigma }^{†}\left(t\right)d_{\sigma }^{†}\left(t^{\prime }\right)\rangle . \end{array}$$

Here *𝒢*_*σ*_(*t*, *t*′) is the QD of two-particle Green's function.

Equations (15) and (18) can be written in a compact form as follows (see Appendix B):

(20) (i∂∂t−ϵηk→σΔησΔηi∂∂t+ϵηk→)(Fηk→σ(t,t′)ℱ~ηk→σ(t,t′)ℱηk→σ(t,t′)F~ηk→σ(t,t′))  =Vησz(Gσ(t,t′)𝒢~ηk→σ(t,t′)𝒢σ(t,t′)G~ηk→σ(t,t′)).

We introduce the tilde Keldysh-Green functions:

(21) $$\begin{array}{c} {\tilde{ℱ}}_{\eta \vec{k}\sigma }\left(t,t^{\prime }\right)=-\text{i}\langle {\text{T}}_{\text{K}}a_{\eta \vec{k}\sigma }\left(t\right)d_{-\sigma }\left(t^{\prime }\right)\rangle ,\\ {\tilde{\text{F}}}_{\eta \vec{k}\sigma }\left(t,t^{\prime }\right)=-\text{i}\langle {\text{T}}_{\text{K}}a_{\eta -\vec{k}-\sigma }^{†}\left(t\right)d_{-\sigma }\left(t^{\prime }\right)\rangle ,\\ {\tilde{𝒢}}_{\sigma }\left(t,t^{\prime }\right)=-\text{i}\langle {\text{T}}_{\text{K}}d_{\sigma }\left(t\right)d_{-\sigma }\left(t^{\prime }\right)\rangle ,\\ {\tilde{\text{G}}}_{\sigma }\left(t,t^{\prime }\right)=-\text{i}\langle {\text{T}}_{\text{K}}d_{-\sigma }^{†}\left(t\right)d_{-\sigma }\left(t^{\prime }\right)\rangle . \end{array}$$

Consider the following 2 × 2 matrix whose elements are the unperturbed Green-Keldysh functions, that is, defined for *V*_*η*_ = 0:

(22) $$\begin{array}{c} \left(\begin{array}{cc} {\text{g}}_{\eta \vec{k}\sigma }\left(t,t^{\prime }\right) & {\text{f}}_{\eta \vec{k}\sigma }\left(t,t^{\prime }\right)\\ {\text{f}}_{\eta \vec{k}\sigma }{\tilde{\text{f}}}_{\eta \vec{k}\sigma }\left(t,t^{\prime }\right) & {\tilde{\text{g}}}_{\eta \vec{k}\sigma }\left(t,t^{\prime }\right) \end{array}\right), \end{array}$$

where

(23) $$\begin{array}{c} {\text{g}}_{\eta \vec{k}\sigma }\left(t,t^{\prime }\right)\equiv -\text{i}{\langle {\text{T}}_{\text{K}}a_{\eta \vec{k}\sigma }\left(t\right)a_{\eta \vec{k}\sigma }^{†}\left(t^{\prime }\right)\rangle }_{0}\\ {\tilde{\text{f}}}_{\eta \vec{k}\sigma }\left(t,t^{\prime }\right)\equiv -\text{i}{\langle {\text{T}}_{\text{K}}a_{\eta \vec{k}\sigma }\left(t\right)a_{\eta -\vec{k}-\sigma }\left(t^{\prime }\right)\rangle }_{0}\\ {\text{f}}_{\eta \vec{k}\sigma }\left(t,t^{\prime }\right)\equiv -\text{i}{\langle {\text{T}}_{\text{K}}a_{\eta -\vec{k}-\sigma }^{†}\left(t\right)a_{\eta \vec{k}\sigma }^{†}\left(t^{\prime }\right)\rangle }_{0}\\ {\tilde{\text{g}}}_{\eta \vec{k}\sigma }\left(t,t^{\prime }\right)\equiv -\text{i}{\langle {\text{T}}_{\text{K}}a_{\eta -\vec{k}-\sigma }^{†}\left(t\right)a_{\eta -\vec{k}-\sigma }\left(t^{\prime }\right)\rangle }_{0}. \end{array}$$

According to Appendix A, their equations of motion are given by

(24) $$\begin{array}{c} \left(\text{i}\frac{\partial }{\partial t}-\epsilon_{\eta \vec{k}}\right){\text{g}}_{\eta \vec{k}\sigma }\left(t,t^{\prime }\right)+\sigma \Delta_{\eta }{\text{f}}_{\eta \vec{k}\sigma }\left(t,t^{\prime }\right)=\delta \left(t,t^{\prime }\right),\\ \left(\text{i}\frac{\partial }{\partial t}-\epsilon_{\eta \vec{k}}\right){\tilde{\text{f}}}_{\eta \vec{k}\sigma }\left(t,t^{\prime }\right)+\sigma \Delta_{\eta }{\tilde{\text{g}}}_{\eta \vec{k}\sigma }\left(t,t^{\prime }\right)=0,\\ \left(\text{i}\frac{\partial }{\partial t}+\epsilon_{\eta \vec{k}}\right){\text{f}}_{\eta \vec{k}\sigma }\left(t,t^{\prime }\right)+\sigma \Delta_{\eta }{\text{g}}_{\eta \vec{k}\sigma }\left(t,t^{\prime }\right)=0,\\ \left(\text{i}\frac{\partial }{\partial t}+\epsilon_{\eta \vec{k}}\right){\tilde{\text{g}}}_{\eta \vec{k}\sigma }\left(t,t^{\prime }\right)+\sigma \Delta_{\eta }{\tilde{\text{f}}}_{\eta \vec{k}\sigma }\left(t,t^{\prime }\right)=\delta \left(t,t^{\prime }\right). \end{array}$$

These equations can be written in matrix form as follows:

(25) (i∂∂t−ϵηk→σΔησΔηi∂∂t+ϵηk→)(gηk→σ(t,t′)f~ηk→σ(t,t′)fηk→σ(t,t′)g~ηk→σ(t,t′))  =(δ(t,t′)00δ(t,t′)).

Equation (20) can be written as an integral along the Keldysh contour C_K_ (for an explanation see Appendix B)

(26) (Fηk→σ(t,t′)ℱ~ηk→σ(t,t′)ℱηk→σ(t,t′)F~ηk→σ(t,t′))  =∫CKdt′′(gηk→σ(t,t′′)f~ηk→σ(t,t′′)fηk→σ(t,t′′)g~ηk→σ(t,t′′))   ×Vησz(Gσ(t′′,t′)𝒢~σ(t′′,t′)𝒢σ(t′′,t′)G~σ(t′′,t′)).

From the last expression one can read for ${\text{F}}_{\eta \vec{k}\sigma }\left(t,t^{\prime }\right)$ the following equation:

(27) Fηk→σ(t,t′) =Vη∫CKdt′′[gηk→σ(t,t′′)Gσ(t′′,t′)       −f~ηk→σ(t,t′′)𝒢σ(t′′,t′)].

We now apply the procedure explained in Appendix C; in order to obtain the ${\text{F}}_{\eta \vec{k}\sigma }(t,t^{\prime })$ lesser component, we obtain

(28) Fηk→σ<(t,t′) =Vη{∫−∞∞dt′′[gηk→σ(r)(t,t′′)Gσ<(t′′,t′)           −f~ηk→σ(r)(t,t′′)𝒢σ<(t′′,t′)]    +∫−∞∞dt′′[gηk→σ<(t,t′′)Gσ(a)(t′′,t′)           −f~ηk→σ <(t,t′′)𝒢σ(a)(t′′,t′)]}.

Furthermore, the superscripts (<), (>), (r), and (a) correspond to lesser, greater, retarded, and advanced Green's functions, respectively.

Therefore, from (12), *I*_*η*_(*t*) can be written as follows:

(29) $$\begin{array}{c} I_{\eta }\left(t\right)=I_{\eta }^{\left(1\right)}\left(t\right)+I_{\eta }^{\left(2\right)}\left(t\right), \end{array}$$

with

(30) Iη(1)(t)=2eℏℜ∑σ∫−∞∞dt′{[Vη2∑k→gηk→σ(r)(t,t′)]Gσ<(t′,t)         +[Vη2∑k→gηk→σ<(t,t′)]Gσ(a)(t′,t)},

(31) Iη(2)(t)=−2eℏℜ∑σ∫−∞∞dt′{[Vη2∑k→f~ηk→σ(r)(t,t′)]𝒢σ<(t′,t)          +[Vη2∑k→f~ηk→σ<(t,t′)]𝒢σ(a)(t′,t)}.

When applying the Fourier transformations, (30) and (31) can be expressed as follows:

(32) Iη(1)(t)=2ehℜ∑σ∫−∞∞dω∫−∞∞dω′2πe−i(ω−ω′)t ×[Ση(r)(ω)Gσ<(ω,ω′)+Ση<(ω)Gσ(a)(ω,ω′)],Iη(2)(t)=−2ehℜ∑σ{e−2iμηt∫−∞∞dω          ×∫−∞∞dω′2πe−i(ω−ω′)t            ×[Ξ~η(r)(ω)σ𝒢σ<(ω,ω′)                +Ξ~η<(ω)σ𝒢σ(a)(ω,ω′)]},

with

(2) $$\begin{array}{c} V_{\eta }^{2}\underset{\vec{k}}{\sum }{\text{g}}_{\eta \vec{k}\sigma }^{\left(\text{r}\right)}\left(t,t^{\prime }\right)\equiv \overset{\infty }{\underset{-\infty }{\int }}\frac{\text{d}\omega }{2\pi }{\text{e}}^{-\text{i}\omega (t-t^{\prime })}\Sigma_{\eta }^{\left(\text{r}\right)}\left(\omega \right),\\ V_{\eta }^{2}\underset{\vec{k}}{\sum }{\text{g}}_{\eta \vec{k}\sigma }^{<}\left(t,t^{\prime }\right)\equiv \overset{\infty }{\underset{-\infty }{\int }}\frac{\text{d}\omega }{2\pi }{\text{e}}^{-\text{i}\omega (t-t^{\prime })}\Sigma_{\eta }^{<}\left(\omega \right),\\ V_{\eta }^{2}\underset{\vec{k}}{\sum }{\tilde{\text{f}}}_{\eta \vec{k}\sigma }^{\left(\text{r}\right)}\left(t,t^{\prime }\right)\equiv \overset{\infty }{\underset{-\infty }{\int }}{\text{e}}^{-2\text{i}\mu_{\eta }t}\sigma \frac{\text{d}\omega }{2\pi }{\text{e}}^{-\text{i}\omega (t-t^{\prime })}{\tilde{\Xi }}_{\eta }^{\left(\text{r}\right)}\left(\omega \right),\\ V_{\eta }^{2}\underset{\vec{k}}{\sum }{\tilde{\text{f}}}_{\eta \vec{k}\sigma }^{<}\left(t,t^{\prime }\right)\equiv \overset{\infty }{\underset{-\infty }{\int }}{\text{e}}^{-2\text{i}\mu_{\eta }t}\sigma \frac{\text{d}\omega }{2\pi }{\text{e}}^{-\text{i}\omega (t-t^{\prime })}{\tilde{\Xi }}_{\eta }^{<}\left(\omega \right). \end{array}$$

In Appendices D to G, we evaluate the unperturbed Green's functions ${\text{g}}_{\eta \vec{k}\sigma }^{\left(\text{r}\right)}\left(t,t^{\prime }\right)$, ${\text{g}}_{\eta \vec{k}\sigma }^{<}\left(t,t^{\prime }\right)$, ${\tilde{\text{f}}}_{\eta \vec{k}\sigma }^{\left(\text{r}\right)}\left(t,t^{\prime }\right)$, and ${\tilde{\text{f}}}_{\eta \vec{k}\sigma }^{<}\left(t,t^{\prime }\right)$ in the wide band limit.

We summarize these results as follows:

(34) $$\begin{array}{c} \Sigma_{\eta }^{\left(\text{r}\right)}\left(\omega \right)=-\Gamma_{\eta }\left[\frac{\omega -\mu_{\eta }}{\Delta_{\eta }}\zeta \left(\Delta_{\eta },\omega -\mu_{\eta }\right)+\text{i}\zeta \left(\omega -\mu_{\eta },\Delta_{\eta }\right)\right],\\ \Sigma_{\eta }^{<}\left(\omega \right)=2\text{i}\Gamma_{\eta }\zeta \left(\omega -\mu_{\eta },\Delta_{\eta }\right)\text{f}\left(\omega -\mu_{\eta }\right),\\ {\tilde{\Xi }}_{\eta }^{\left(\text{r}\right)}\left(\omega \right)=\Gamma_{\eta }\left[\zeta \left(\Delta_{\eta },\omega +\mu_{\eta }\right)+\text{i}\frac{\Delta_{\eta }}{\omega +\mu_{\eta }}\zeta \left(\omega +\mu_{\eta },\Delta_{\eta }\right)\right],\\ {\tilde{\Xi }}_{\eta }^{<}\left(\omega \right)=-2\text{i}\Gamma_{\eta }\frac{\Delta_{\eta }}{\omega +\mu_{\eta }}\zeta \left(\omega +\mu_{\eta },\Delta_{\eta }\right)\text{f}\left(\omega +\mu_{\eta }\right),\\ \zeta \left(\omega ,\omega^{\prime }\right)\equiv \Theta \left(\left|\omega \right|-\left|\omega^{\prime }\right|\right)\frac{\left|\omega \right|}{\sqrt{\omega^{2}-\omega^{\prime 2}}}. \end{array}$$

All these expressions will used below.

## 3. QD Green Function

We need to evaluate the most important objet for calculations, namely, QD Green's functions given by (17) and (19), as well as their respective tilde functions:

(35) $$\begin{array}{c} {\tilde{\text{G}}}_{\sigma }\left(t,t^{\prime }\right)=-\text{i}\langle {\text{T}}_{\text{K}}d_{-\sigma }^{†}\left(t\right)d_{-\sigma }\left(t^{\prime }\right)\rangle ,\\ {\tilde{𝒢}}_{\sigma }\left(t,t^{\prime }\right)=-\text{i}\langle {\text{T}}_{\text{K}}d_{\sigma }\left(t\right)d_{-\sigma }\left(t^{\prime }\right)\rangle . \end{array}$$

Again using the scheme given in Appendix A, their equation of motion is

(36) i∂∂t(Gσ(t,t′)𝒢~σ(t,t′)𝒢σ(t,t′)G~σ(t,t′)) =(δ(t,t′)−i〈TK[dσ(t),H]dσ†(t′)〉−i〈TK[dσ(t),H]d−σ(t′)〉−i〈TK[d−σ†(t),H]dσ†(t′)〉δ(t,t′)−i〈TK[d−σ†(t),H]d−σ(t′)〉),

which develops to

(37) i∂∂tGσ(t,t′)=δ(t,t′)−iE0〈TKdσ(t)dσ†(t′)〉 −i∑ηk→Vη〈TKaηk→σ(t)dσ†(t′)〉=δ(t,t′)+E0Gσ(t,t′)+∑ηk→VηFηk→σ(t,t′),i∂∂t𝒢σ(t,t′)=iE0〈TKd−σ†(t)dσ†(t′)〉 +i∑ηk→Vη〈TKaη−k→−σ†(t)dσ†(t′)〉=−E0𝒢σ(t,t′)−∑ηk→Vηℱηk→σ(t,t′),i∂∂t𝒢~σ(t,t′)=−iE0〈TKdσ(t)d−σ(t′)〉 −i∑ηk→Vη〈TKaηk→σ(t)d−σ(t′)〉=E0𝒢~σ(t,t′)+∑ηk→Vηℱ~ηk→σ(t,t′),i∂∂tG~σ(t,t′)=δ(t,t′)+iE0〈TKd−σ†(t)d−σ(t′)〉 +i∑ηk→Vη〈TKaηk→σ†(t)d−σ(t′)〉=δ(t,t′)−E0G~σ(t,t′)−∑ηk→Vηℱ~ηk→σ(t,t′).

This can be written as follows:

(38) (i∂∂t−E000i∂∂t+E0)(Gσ(t,t′)𝒢~σ(t,t′)𝒢σ(t,t′)G~σ(t,t′))  =(δ(t,t′)00δ(t,t′))   +∑ηk→Vησz(Fηk→σ(t,t′)ℱ~ηk→σ(t,t′)ℱηk→σ(t,t′)F~ηk→σ(t,t′)).

When *V*_*η*_ = 0, one has

(39) (i∂∂t−E000i∂∂t+E0)(G0(t,t′)00G~0(t,t′))  =(δ(t,t′)00δ(t,t′)),

with

(40) $$\begin{array}{c} {\text{G}}_{0}\left(t,t^{\prime }\right)=-\text{i}{\langle {\text{T}}_{\text{K}}d_{\sigma }\left(t\right)d_{\sigma }^{†}\left(t^{\prime }\right)\rangle }_{0},\\ {\tilde{\text{G}}}_{0}\left(t,t^{\prime }\right)=-\text{i}{\langle {\text{T}}_{\text{K}}d_{-\sigma }^{†}\left(t\right)d_{-\sigma }\left(t^{\prime }\right)\rangle }_{0},\\ {\text{G}}_{0}\left(t,t^{\prime }\right)\equiv {{\text{G}}_{\sigma }\left(t,t^{\prime }\right)|}_{V_{\eta }=0},\\ {\tilde{\text{G}}}_{0}\left(t,t^{\prime }\right)\equiv {{\tilde{\text{G}}}_{\sigma }\left(t,t^{\prime }\right)|}_{V_{\eta }=0}. \end{array}$$

The last two equations can be written as follows:

(41) (i∂∂t−E000i∂∂t+E0)  ×(Gσ(t,t′)−G0(t,t′)𝒢~σ(t,t′)𝒢σ(t,t′)G~σ(t,t′)−G~0(t,t′)) =∑ηk→Vησz(Fηk→σ(t,t′)ℱ~ηk→σ(t,t′)ℱηk→σ(t,t′)F~ηk→σ(t,t′)).

We write the last equation in its equivalent convolution integral along the Keldysh contour (see Appendix B):

(42) (Gσ(t,t′)−G0(t,t′)𝒢~σ(t,t′)𝒢σ(t,t′)G~σ(t,t′)−G~0(t,t′))  =∫CKdt′′(G0(t,t′′)00G~0(t,t′′))   ×∑ηk→Vησz(Fηk→σ(t′′,t′)ℱ~ηk→σ(t′′,t′)ℱηk→σ(t′′,t′)F~ηk→σ(t′′,t′)).

An equivalent way to write the last equation (using (25)) as a convolution of Σ_*σ*_(*t*, *t*′) and **G**_*σ*_(*t*, *t*′) is

(43) Gσ(t,t′) =G0(t,t′)+∫CKdt′′G0(t,t′)Σσ(t′,t′′)Gσ(t′′,t′),

with

(44) Gσ(t,t′)≡(Gσ(t,t′)𝒢~σ(t,t′)𝒢σ(t,t′)G~σ(t,t′)),G0(t,t′)≡(G0(t,t′)00G~0(t,t′)),Σσ(t,t′) ≡∫CKdt′′(Vη2∑ηk→gηk→σ(t′′,t′)−Vη2∑ηk→f~ηk→σ(t′′,t′)−Vη2∑ηk→fηk→σ(t′′,t′)Vη2∑ηk→g~ηk→σ(t′′,t′)).

We are interested in two regimes: a first regime in which *U*_0_ ~ Γ < Δ and the Coulomb blockade effects are neglected because in this case the couplings to the leads are not extremely small and the dot capacitance is large enough, a second regime for *U*_0_ ~ Δ > Γ where Coulomb blockade effects must be taken into account. For both regimes and from now on, we are interested in the case *eV* > Δ, where multiple Andreev reflection [42] processes are strongly suppressed. Therefore only the single particle current (SP) has to be considered *I*_SP_. From the above considerations, we have that the Keldysh Green function *𝒢*_*σ*_(*ω*), which carries information of the quantum dot two-particle Green's function, can be neglected and all relevant information is contained in G_*σ*_(*ω*).

The Keldysh Green function becomes spin independent; G_*σ*_(*ω*) ≡ G(*ω*). Element 11 of (43) is given by

(45) G(t,t′)=G0(t,t′)+∫CKdt′′∫CKdt′′′G0(t,t′′) ×Σ(t′′,t′′′)G(t′′′,t′).

Again, using the recipe given in Appendix C, we obtain for G^<^(*t*, *t*′) and G^(a)^(*t*, *t*′) the following:

(46) G<(t,t′) =G0<(t,t′)  +[∫−∞∞dt′′∫−∞∞dt′′′G0(r)(t,t′′)          ×Σ(r)(t′′,t′′′)G<(t′′′,t′)    +G0(r)(t,t′′)Σ<(t′′,t′′′)     ×G(a)(t′′′,t′)+G0<(t,t′′)     ×Σ(a)(t′′,t′′′)G(a)(t′′′,t′)],G(a)(t,t′) =G0(a)(t,t′)  +∫−∞∞dt′′∫−∞∞dt′′′G0(a)(t,t′′)Σ(a)(t′′,t′′′)G(a)(t′′′,t′).

Taking the Fourier transform of (46) results in a set of algebraic equations:

(47) G<(ω,ω′) =2πδ(ω−ω′)G0<(ω)+G0(r)(ω)Σ(r)(ω)G<(ω,ω′)  +G0(r)(ω)Σ<(ω)G(a)(ω,ω′)+G0<(ω)Σ(a)(ω)  ×G(a)(ω,ω′),G(a)(ω,ω′) =2πδ(ω−ω′)G0(a)(ω)+G0(a)(ω)Σ(a)(ω)G(a)(ω,ω′).

Dot Keldysh Green's functions G_*σ*_^<^(*ω*, *ω*′) and G_*σ*_^(a)^(*ω*, *ω*′) are below straightforward evaluated. In this regime, quantities such as currents are independent of time. Therefore, we have

(48) $$\begin{array}{c} {\text{G}}^{<}\left(\omega ,\omega^{\prime }\right)=2\pi \delta \left(\omega -\omega^{\prime }\right){\text{G}}^{<}\left(\omega \right),\\ {\text{G}}^{\left(\text{a}\right)}\left(\omega ,\omega^{\prime }\right)=2\pi \delta \left(\omega -\omega^{\prime }\right){\text{G}}^{\left(\text{a}\right)}\left(\omega \right). \end{array}$$

Therefore (47) result in

(49) G<(ω)=G0<(ω)+G0(r)(ω)Σ(r)(ω)G<(ω) +G0(r)(ω)Σ<(ω)G(a)(ω) +G0<(ω)Σ(a)(ω)G(a)(ω),

(50) $$\begin{array}{c} {\text{G}}^{\left(\text{a}\right)}\left(\omega \right)={\text{G}}_{0}^{\left(\text{a}\right)}\left(\omega \right)+{\text{G}}_{0}^{\left(\text{a}\right)}\left(\omega \right)\Sigma^{\left(\text{a}\right)}\left(\omega \right){\text{G}}^{\left(\text{a}\right)}\left(\omega \right). \end{array}$$

Solving (50),

(51) G(a)(ω)=1G0(a)(ω)−1−Σ(a)(ω)=1ω−E0−Σ(a)(ω)=G(r)(ω)∗.

Moreover, we know that

(52) $$\begin{array}{c} {\text{G}}_{0}^{<}\left(\omega \right)\propto \delta \left(\omega -E_{0}\right),\\ {\text{G}}_{0}^{\left(\text{a}\right)}\left(\omega \right)={\left(\omega -E_{0}-\text{i}0^{+}\right)}^{-1}, \end{array}$$

Resulting in

(53) $$\begin{array}{c} {\text{G}}_{0}^{<}\left(\omega \right)\Sigma^{\left(\text{a}\right)}\left(\omega \right){\text{G}}^{\left(\text{a}\right)}\left(\omega \right)=-{\text{G}}_{0}^{<}\left(\omega \right). \end{array}$$

Equation (49) is reduced to

(54) $$\begin{array}{c} {\text{G}}^{<}\left(\omega \right)={\text{G}}_{0}^{\left(\text{r}\right)}\left(\omega \right)\Sigma^{\left(\text{r}\right)}\left(\omega \right){\text{G}}^{<}\left(\omega \right)+{\text{G}}_{0}^{\left(\text{r}\right)}\left(\omega \right)\Sigma^{<}\left(\omega \right){\text{G}}^{\left(\text{a}\right)}\left(\omega \right), \end{array}$$

(55) G<(ω)=Σ<(ω)G(a)(ω)G0(r)(ω)−1−Σ(r)(ω)=Σ<(ω)|G(r)(ω)|2=πΣ<(ω)−ℑG(r)(ω)/πℑ(G(r)(ω))−1=πΣ<(ω)−ℑΣ(r)(ω)ρ(ω).

Here *ρ*(*ω*) is the so-called quantum dot spectral function which is given in terms of the imaginary part (*ℑ*) of the retarded Keldysh Green function G^(r)^(*ω*):

(56) ρ(ω)=−1πℑG(r)(ω)=−1πℑΣ(r)(ω)ω−E0−ℜΣ(r)(ω)2+ℑΣ(r)(ω)2.

From (30), the single particle current (*I*_SP_) results in

(57) IηSP(V,E0)=4ehℜ∫−∞∞dω[Ση(r)(ω)G<(ω)         +Ση<(ω)G(a)(ω)].

Substituting (51) and (55) in (57),

(58) IηSP(V,E0)  =4eh∫−∞∞dω[πℑΣη(r)(ω)ℑΣ<(ω)ℑΣ(r)(ω)ρ(ω)          +ℑΣη<(ω)ℑG(r)(ω)]  =4πeh∫−∞∞dωρ(ω)[ℑΣη(r)(ω)ℑΣ<(ω)ℑΣ(r)(ω)ρ(ω)              −ℑΣη<(ω)]  =4πeh∫−∞∞dωρ(ω)Γ(ω)[Γη(ω)ℑΣ<(ω)              −Γ(ω)ℑΣη<(ω)],

with Γ_*η*_(*ω*) = −*ℑ*Σ_*η*_^(r)^(*ω*) = Γ_*η*_ *ζ*(*ω*, Δ_*η*_) and Γ(*ω*) = ∑_*η*_Γ_*η*_(*ω*). In our regime, *eV* > Δ; therefore, *ℜ*Σ^(r)^(*ω*) in the above equations is zero. We use the expression for Σ^(r)^(*ω*) from Appendix D and obtain the single particle current *I*_SP_ ≡ (*I*_*R*,SP_ − *I*_*L*,SP_)/2:

(59) ISP(V,E0)=8πeh∫−∞∞dωΓL(ω)ΓR(ω+eV)ΓL(ω)+ΓR(ω+eV)     ×ρ(ω)[f(ω)−f(ω+eV)].

−*eV* = *μ*_*L*_ − *μ*_*R*_ corresponds to the applied voltage between the superconductors electrodes with chemical potential *μ*_*η*_. In the following, we fix the chemical potential *μ*_*L*_ = 0 and use *eV* as a measure of *μ*_*R*_. In addition, the QD energy *E*_0_ is measured with respect to *μ*_*L*_. On the other hand, the limits of integration are given by the functions Γ_*L*_(*ω*) and Γ_*R*_(*ω* + *eV*). The extra 2*π* factor arises from the dot Keldysh Green functions. *ρ*(*ω*) and Γ(*ω*) are given by

(60) $$\begin{array}{c} \rho \left(\omega \right)=\frac{\Gamma \left(\omega \right)/\pi }{{\left(\omega -E_{0}\right)}^{2}+\Gamma^{2}\left(\omega \right)}, \end{array}$$

(61) $$\begin{array}{c} \Gamma \left(\omega \right)=\Gamma_{L}\left(\omega \right)+\Gamma_{R}\left(\omega +eV\right). \end{array}$$

At steady state there is no net flow into or out of the mesoscopic channel or quantum dot which yields a stationary particle number in it. The population number *N*, at the dot, is given by

(62) $$\begin{array}{c} N=2\left[-\text{i}{\text{G}}^{<}\left(t,t\right)\right]=2\overset{\infty }{\underset{-\infty }{\int }}\frac{\text{d}\omega }{2\pi \text{i}}{\text{G}}^{<}\left(\omega \right), \end{array}$$

which becomes a weighted average over the *L* and *R* contacts:

(63) $$\begin{array}{c} N=2\overset{\infty }{\underset{-\infty }{\int }}\text{d}\omega \rho \left(\omega \right)\left[\frac{\Gamma_{L}\left(\omega \right)}{\Gamma \left(\omega \right)}\text{f}\left(\omega \right)+\frac{\Gamma_{R}\left(\omega +eV\right)}{\Gamma \left(\omega \right)}\text{f}\left(\omega +eV\right)\right]. \end{array}$$

For the N/QD/N case, Γ_*R*,*L*_ are just constants. This case was studied in the context of the generalized quantum master approach (section IV in [39]). That approach permits the inclusion of broadening in a natural way. They obtained Equations similar to (59)–(63).

## 4. Coupled Poisson Nonequilibrium Green Function Scheme: The Capacitive Model

So far, we are not including the side effects of a potential profile inside the mesoscopic channel. On the one hand, its inclusion takes in order zero or Hartree approximation the electron-electron interaction in the QD. Its inclusion also guarantees current independence from the choice of zero potential [32]. Such potential is induced by the action of source, drain and gate applied voltages. In principle, we have to couple the number of population equations. Equation (62), with electric field *U*. However, since the number of quantum levels in the channel is small and the particle number variation is negligible, the potential profile variation inside the channel is negligible. Then it is appropriate to visualize the channel as an equivalent circuit framework (Figure 2). In this framework, we associate capacitances *C*_*d*_, *C*_*s*_, and *C*_*g*_ with the drain, source and gate, respectively. Whenever drain, source, and gate bias potentials *V*_*d*_, *V*_*s*_, and *V*_*g*_, respectively, are present, there is an electrostatic potential *V*_QD_ inside the QD, which induces an energy shift of the QD energy level *U* = −*e*(*V*_QD_ − *V*_0_), *V*_0_ are channel electrostatic potential before we apply the source and drain biases, respectively.

The electronic populations before and after we apply the biases mentioned above are given by

(64) −eN0=CdV0+CsV0+CgV0,−eN=Cd(VQD−Vd)+Cs(VQD−Vs) +Cg(VQD−Vg),

respectively. It leads us to

(65) −eΔN≡−e(N−N0)=CE(VQD−V0) −CdVd−CsVs−CgVg,

where *C*_*E*_ = *C*_*d*_ + *C*_*s*_ + *C*_*g*_. Therefore, the energy shift *U* is given by

(66) $$\begin{array}{c} U=U_{ℒ}+\frac{e^{2}}{C_{E}}\Delta N, \end{array}$$

where

(67) $$\begin{array}{c} U_{ℒ}\equiv \frac{C_{d}}{C_{E}}\left(-eV_{d}\right)+\frac{C_{s}}{C_{E}}\left(-eV_{s}\right)+\frac{C_{g}}{C_{E}}\left(-eV_{g}\right). \end{array}$$

In the expression for *U*, *U*_*ℒ*_ represents a uniform shift for all levels, whereas the second term (the Poisson contribution denoted by *U*_*P*_ in the introduction) represents a level of repulsion which is proportional to the averaged occupation of the QD level denoted by *N*_0_ and proportional to the charging energy *U*_0_ = *e* ^2^/*C*_*E*_.

On the other hand, one has Δ*N* from (62) and (65) given by

(68) $$\begin{array}{c} \Delta N=2\overset{\infty }{\underset{-\infty }{\int }}\frac{\text{d}\omega }{2\pi \text{i}}\left[{\text{G}}^{<}\left(\omega ,U\right)-{\text{G}}^{<}\left(\omega ,-eV_{0}\right)\right]. \end{array}$$

In the expression for G^<^(*ω*, *U*) (see (55)), the energy level shifts only (*E*_0_⇒(*E*_0_ + *U*)) in the expression for the QD spectral function *ρ*(*ω* − *U*). Equations (66) and (68) are coupled nonlinear equations with unknowns *U* and Δ*N*. We solve the coupled equations via an iteration procedure. First we guest a value for Δ*N* plug this value in *U*, and then we calculate Δ*N* with the following equation:

(69) ΔN=2∫−∞∞dωρ(ω−U)    ×ΓL(ω)f(ω)+ΓR(ω+eV)f(ω+eV)ΓL(ω)+ΓR(ω+eV),

and so on until convergence is achieved. With the final value of *U* obtained for a given bias voltage *V*, *I*_SP_ is calculated via the equation:

(70) ISP(V,U)=8πeh∫−∞∞dωΓL(ω)ΓR(ω+eV)ΓL(ω)+ΓR(ω+eV)     ×ρ(ω−U)[f(ω)−f(ω+eV)].

In summary, the procedure for computing *I* consists of the following steps. (i) Determine the spectral density. (ii) Specify *V*_*g*_, *V*_*d*_, *V*_*s*_, and coupling constants. (iii) Iteratively solve (69) and (66). (iv) Evaluate the current from (70) for the *V*_*g*_, *V*_*d*_, and *V*_*s*_. Once a converged *U* has been found, the current is finally evaluated.

The way we consider electron-electron interactions imposes restrictions on the possible values of the charging energy *U*_0_. For the self-consistent scheme to be valid, we have to assume that Δ ≫ Γ_*L*,*R*_≃*U*_0_. However, less precisely quantitative results, although qualitative correct results can be obtained if Δ≃*U*_0_ ≫ Γ_*L*,*R*_, when the so-called Coulomb Blockade energy dominates over the coupling constants. For this case, we use the improvement of the SCF method discussed in the introduction [4, 29, 30]. The self-consistent generalizes to

(71) $$\begin{array}{c} U_{↑}=U_{ℒ}+\frac{e^{2}}{C_{E}}\left(N_{↓}-N_{0}\right), \end{array}$$

(72) $$\begin{array}{c} U_{↓}=U_{ℒ}-\frac{e^{2}}{C_{E}}\left(N_{↑}-N_{0}\right), \end{array}$$

where the up-spin level feels a potential due to the down-spin electrons and viceversa. Notice the different signs which reflects the Coulomb repulsion between otherwise degenerate levels:

(73) N↑=∫−∞∞dωρ(ω−U↑)   ×ΓL(ω)f(ω)+ΓR(ω+eV)f(ω+eV)ΓL(ω)+ΓR(ω+eV),

(74) N↓=∫−∞∞dωρ(ω−U↓)   ×ΓL(ω)f(ω)+ΓR(ω+eV)f(ω+eV)ΓL(ω)+ΓR(ω+eV),

(75) $$\begin{array}{c} N=N_{↑}+N_{↓}. \end{array}$$

Here, *N*_↑_ and *N*_↓_ are the population of the spin-up and spin-down levels. Once the values of *U*_↑_ and *U*_↓_ are calculated, *I*_SP_ is calculated from

(76) ISP(V,U↑,U↓) =4πeh∫−∞∞dωΓL(ω)ΓR(ω+eV)ΓL(ω)+ΓR(ω+eV)      ×{ρ(ω−U↑)+ρ(ω−U↓)}      ×[f(ω)−f(ω+eV)].

As Datta has pointed out [4], the approach described above (called unrestricted SCF) can lead to a better quantitative agreement in comparison with a conceptually correct multi-level Master equation calculation.

## 5. Numerical Results and Remarks on Experiments

### 5.1. First Case: Δ ≫ Γ_*L*,*R*_≃*U*_0_

**Figure 3:**
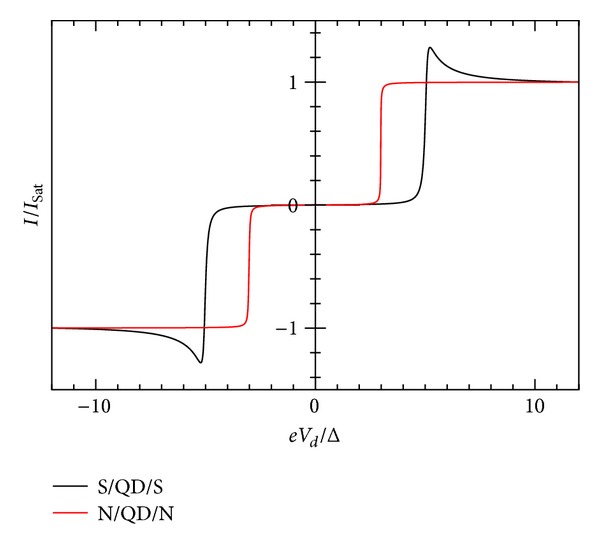
Zero temperature *I*-*V* characteristics for superconductor-quantum-dot-superconductor system, calculated using the self-consistent field (SCF) method, with *E*_0_ = 1.5Δ, *eV*_*g*_ = 0.0Δ, *U*_0_ = 0.005Δ, *C*_*d*_/*C*_*E*_ = 0.5, and Γ_*L*_ = Γ_*R*_ = 0.005Δ.

**Figure 4:**
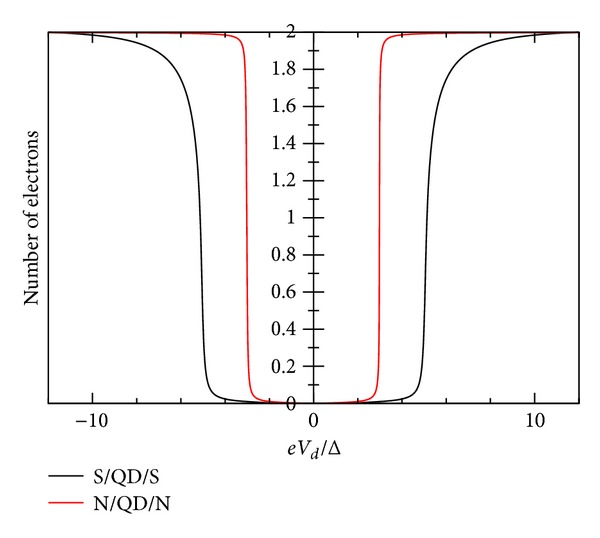
Zero temperature number of electrons *eV*_*d*_/Δ graph for superconductor-quantum-dot-superconductor system, calculated using the self-consistent field (SCF) method, with *E*_0_ = 1.5Δ, *eV*_*g*_ = 0.0Δ, *U*_0_ = 0.005Δ, *C*_*d*_/*C*_*E*_ = 0.5, and Γ_*L*_ = Γ_*R*_ = 0.005Δ.

In this regime, there are the multiple Andreev reflections [42] for voltages such that *eV* < Δ (MAR). Also, there is the possibility for quasiparticle co-tunneling current for energy levels far from *μ*_*L*_. These cases will be considered in a future work that involves the whole expressions we have derived for the currents (see (30) and (31)) and eventually more accurate Green-Keldysh functions and the use of master equations [17]. This case was studied experimentally in [43]. For given values of capacitances and source voltage, we iterate (66) and (68) in order to find the potential *U*. Then the single particle current *I*_SP_(*V*, *U*) is evaluated (see (70)). We put the charge before biasing *N*_0_ = 0, such that Coulomb repulsion with the QD-energy level is absent. Anyhow, in this regime, the effect of the second term in (74) is negligible. Consequently, the Laplace term *U*_*ℒ*_ essentially positions the QD degenerate energy level (with respect to *μ*_*L*_ = 0). In Figure 3, we show *I*-*V* characteristic for gate voltage values *V*_*g*_ = 0 and *K*_*B*_ *T* = Δ, whereas Figure 4 shows the occupation number Δ*N*. These curves are symmetric, due to the assumed equality of the coupling capacitances (*C*_*d*_/*C*_*E*_ = 0.5). Otherwise, the *I*-*V* shifts to right or to the left for *C*_*d*_/*C*_*E*_ = 0.5 > 0.5 or <0.5 respectively. For this case, we show in Figure 7 the spectral density *ρ*(*ω*) for *eV*_*d*_ = −6Δ. Notice that the position of the energy level is essentially −4.5Δ, that is, just the sum of *E*_0_ + *U*_*ℒ*_. Qualitatively, these results are similar to Levy Yeyati et al.'s [17]. Characteristic is the broadening of the BSC singularity. The effect of bigger values of Γ_*R*,*L*_ is a more pronounced round off the BCS-type singularity. We discuss this issue below. For large enough bias, the current approaches the normal saturation value I_Sat_.

### 5.2. Second Case: Δ≃*U*_0_ ≫ Γ_*L*,*R*_

**Figure 5:**
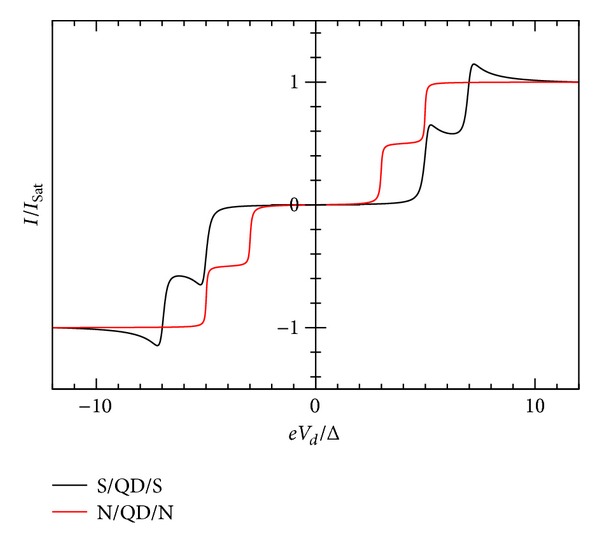
Zero temperature *I*-*V* characteristic showing the Coulomb blockade for superconductor-quantum-dot-superconductor system, calculated using the self-consistent field (SCF) method, with *E*_0_ = 1.5Δ, *eV*_*g*_ = 0.0Δ, *U*_0_ = 1.0Δ, *C*_*d*_/*C*_*E*_ = 0.5, and Γ_*L*_ = Γ_*R*_ = 0.01Δ.

**Figure 6:**
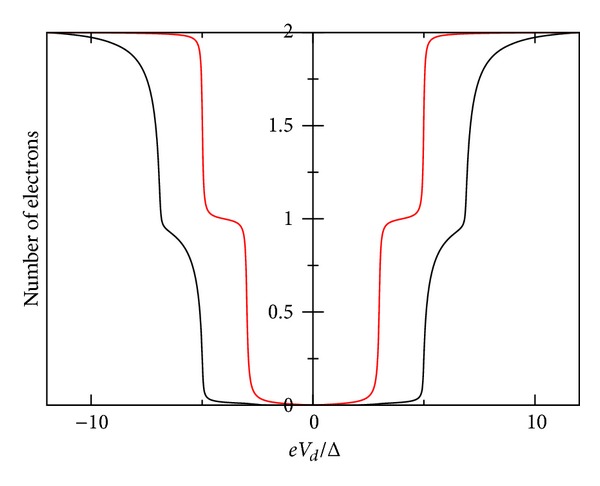
Zero temperature number of electrons-*eV*_*d*_/Δ for superconductor-quantum-dot-superconductor system, calculated using the self-consistent field (SCF) method, with *E*_0_ = 1.5Δ, *eV*_*g*_ = 0.0Δ, *U*_0_ = 1.0Δ, *C*_*d*_/*C*_*E*_ = 0.5, and Γ_*L*_ = Γ_*R*_ = 0.01Δ.

**Figure 7:**
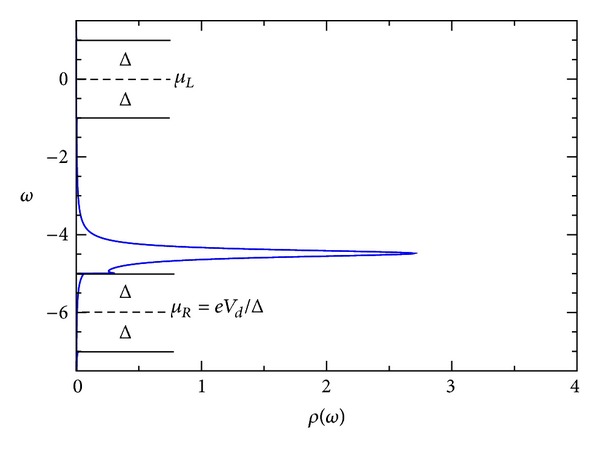
Spectral density of the quantum dot-*ω* for superconductor-quantum-dot-superconductor system, calculated using the self-consistent field (SCF) method, with *E*_0_ = 1.5Δ, *eV*_*g*_ = 0.0Δ, *eV*_*d*_ = 6.0Δ, *U*_0_ = 0.01Δ, *C*_*d*_/*C*_*E*_ = 0.5, and Γ_*L*_ = Γ_*R*_ = 0.05Δ.

**Figure 8:**
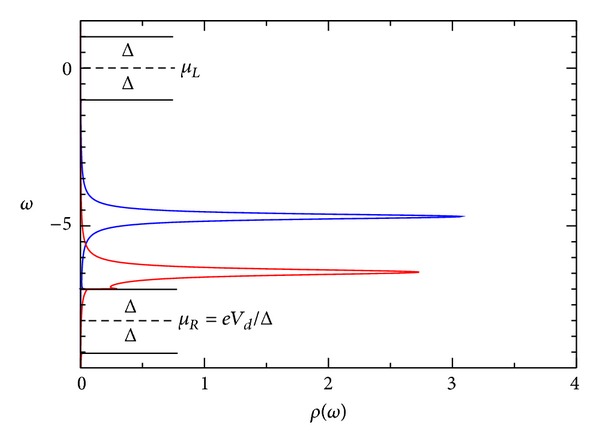
Spectral density of the quantum dot-*ω* for superconductor-quantum-dot-superconductor system, calculated using the self-consistent field (SCF) method, with *E*_0_ = 1.5Δ, *eV*_*g*_ = 0.0Δ, *eV*_*d*_ = 8.0Δ, *U*_0_ = 1.0Δ, *C*_*d*_/*C*_*E*_ = 0.5, and Γ_*L*_ = Γ_*R*_ = 0.20Δ.

In this regime, the charging energy acts effectively in lifting the degeneracy of the otherwise single degenerate QD energy level. For this regime, we use the couple system defined by (71)–(75) and calculate the current according to (76). This is the unrestricted SCF method mentioned in the introduction. The transport begins through one level as long as there is in average less than one electron in it. For the given parameters, the onset of current is similar to the first case (no interaction with residual charge in the QD is considered). However, when the average occupation exceeds one, the other degenerate levels float according to the resulting values *U*_↑_ and *U*_↓_. These values push down the position of this second level and push up the already occupied energy level. In Figures 5-6, we show *I*-*V* and the number of electrons. In Figure 8, it is shown that the spectral density for *eV*_*d*_ = −8Δ. In this case, *E*_0_ + *U*_*ℒ*_ = −5.5Δ. The values obtained from the SCF calculation position the energy levels to −4.846Δ and to −6.396Δ (see (72)).

The experimental work of Ralph et al. [28] corresponds to this second case. In their Figure 3, they show the current for single state level peaks *I* ~ 5 pA at a bias *V* ~ 2.4 mV. According to Figures 5-6, for this sample at 30 mK, there should be another peak at *V* ~ 4.4 mV with a current value *I*_SP_≃10 pA. The charging energy for this sample is *U*_0_≃2.0 mV. However, in their samples with radios ~2.5 nm or greater, the level spacing of the energy levels is such, that Δ*E* < *E*_*c*_; therefore another current signal may occur before and a quantitative proper description would be a multilevel QD-model. One notice, however, the strong fluctuations in the spacing in Figure 2 [28], indicating complex charging for many levels of phenomena. Notice that the theoretical explanation of Levy Yeyati et al. [17, 18] does not contain our prediction.

### 5.3. Influence of Coupling Constants

**Figure 9:**
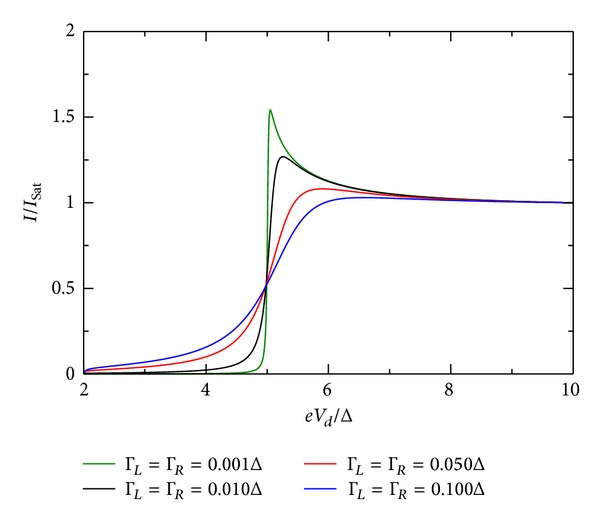
Zero temperature *I*-*V* characteristic for superconductor-quantum-dot-superconductor system for various values of the coupling Γ, calculated using the self-consistent field (SCF) method, with *E*_0_ = 1.5Δ, *eV*_*g*_ = 0.0Δ, *U*_0_ = 0.05Δ, *C*_*d*_/*C*_*E*_ = 0.5, and Γ_*L*_ = Γ_*R*_.

**Figure 10:**
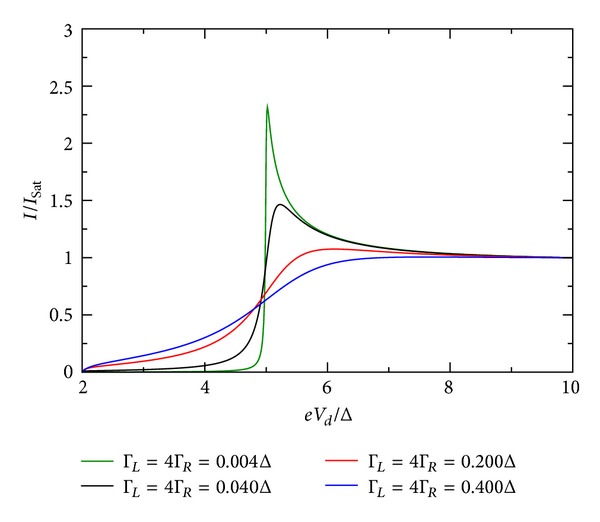
Zero temperature *I*-*V* characteristic for superconductor-quantum-dot-superconductor system for various values of the coupling Γ, calculated using the self-consistent field (SCF) method, with *E*_0_ = 1.5Δ, *eV*_*g*_ = 0.0Δ, *U*_0_ = 0.05Δ, *C*_*d*_/*C*_*E*_ = 0.5, and Γ_*L*_ = 4Γ_*R*_.

**Figure 11:**
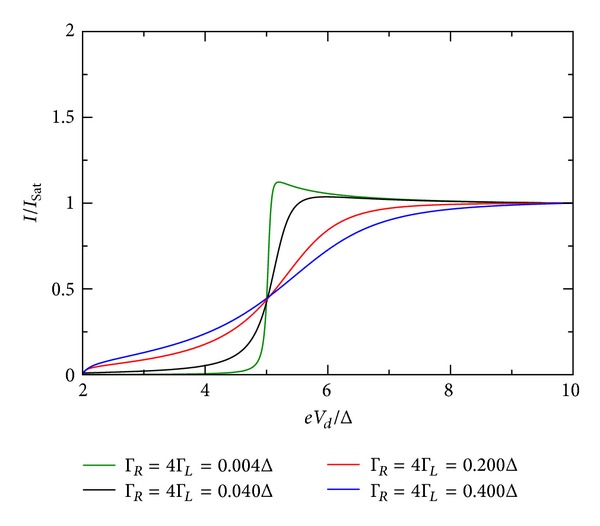
Zero temperature *I*-*V* characteristic for superconductor-quantum-dot-superconductor system for various values of the coupling Γ, calculated using the self-consistent field (SCF) method, with *E*_0_ = 1.5Δ, *eV*_*g*_ = 0.0Δ, *U*_0_ = 0.05Δ, *C*_*d*_/*C*_*E*_ = 0.5, and Γ_*R*_ = 4Γ_*L*_.

**Figure 12:**
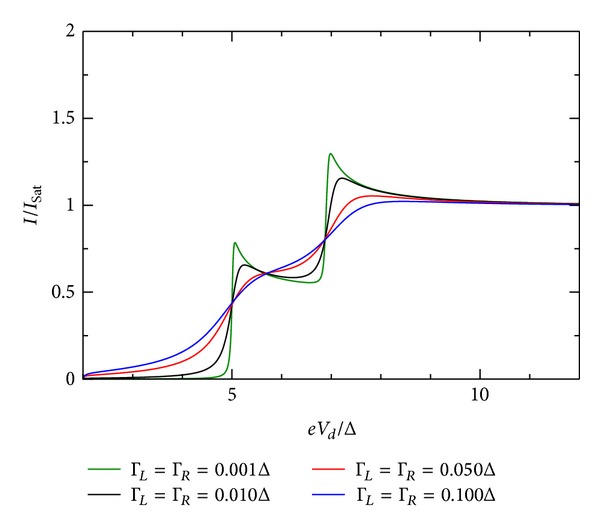
Zero temperature *I*-*V* characteristic for superconductor-quantum-dot-superconductor system for various values of the coupling Γ, calculated using the self-consistent field (SCF) method, with *E*_0_ = 1.5Δ, *eV*_*g*_ = 0.0Δ, *U*_0_ = 1.0Δ, *C*_*d*_/*C*_*E*_ = 0.5, and Γ_*L*_ = Γ_*R*_.

**Figure 13:**
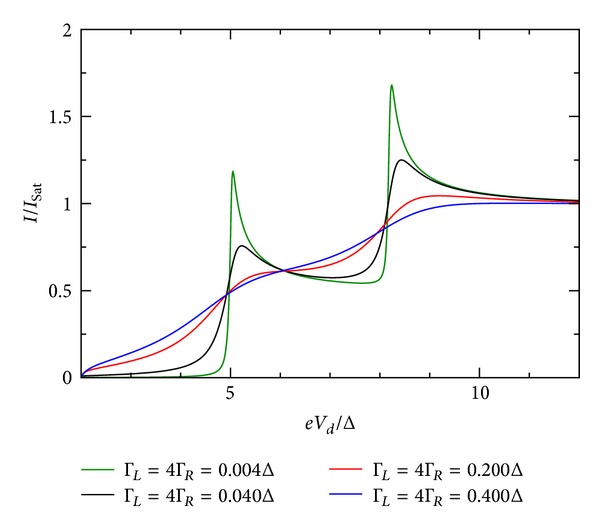
Zero temperature *I*-*V* characteristic for superconductor-quantum-dot-superconductor system for various values of the coupling Γ, calculated using the self-consistent field (SCF) method, with *E*_0_ = 1.5Δ, *eV*_*g*_ = 0.0Δ, *U*_0_ = 1.0Δ, *C*_*d*_/*C*_*E*_ = 0.5, and Γ_*L*_ = 4Γ_*R*_.

**Figure 14:**
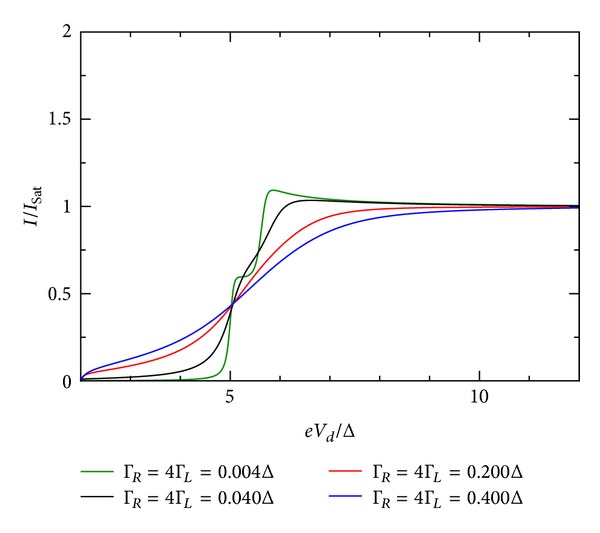
Zero temperature *I*-*V* characteristic for superconductor-quantum-dot-superconductor system for various values of the coupling Γ, calculated using the self-consistent field (SCF) method, with *E*_0_ = 1.5Δ, *eV*_*g*_ = 0.0Δ, *U*_0_ = 1.0Δ, *C*_*d*_/*C*_*E*_ = 0.5, and Γ_*R*_ = 4Γ_*L*_.

The influence of the coupling constant is to broaden the otherwise sharp energy QD level. However, the broadening is not equally strong and depends on the relative values of Γ_*R*_/Γ_*L*_. Ralph et al. [28] determined for the sample in his Figure 3  Γ_*R*_/Γ_*L*_≫1.  Figure 10 shows the *I*-*V* characteristic for the restricted case when both coupling constants are equal. For larger values of the coupling constants, the broadening is stronger. If they are dissimilar in value, the broadening is stronger when Γ_*R*_ > Γ_*L*_. This effect is shown in Figures 9, 10, 11, 12, 13, and 14. This effect is due to the stronger involving of the BSC-DOS singularity of the left lead in the integral expression for the current (see (59)) and the particular choice of the zero bias voltage (see Figure 2).

## 6. Summary and Perspectives

We have studied the single particle current through a quantum dot coupled with two superconductor leads via a coupled Poisson Nonequilibrium Green function (PNEGF) formalism. In a systematic and self-contained way, we derived the expressions for the current in full generality. In this work we focused only on the weak coupling regime where single particle current is the dominant one. The QD is a single degenerate energy level system modeled via a capacitive circuit. The influence of the potential on the QD and on the *I*-*V* characteristic is calculated for relevance values of the coupling and capacitances and the implication of experiments is discussed. This was done in the weak coupling regime and for Δ ≫ Γ_*L*,*R*_≃*U*_0_. A second case when Δ≃*U*_0_ ≫ Γ_*L*,*R*_ also in the weak coupling regime was analyzed. Admittedly, our model of a hybrid system S/QD/S possesses potentially physical extensions. One important missed point is dephasing. This physical effect due to scattering of transport electrons can be incorporated in the self energy phenomenologically [44, 45], or in a stochastic fashion [46]. Another point is to consider a QD with many energy levels and within the self-consistent scheme, to consider the strong and intermediate regimes and many body correlations due to different kinds of electron-electron interaction. Here we have to notice that it is not just to scale the level spacing by the charging energy [47]. It is a genuine many body problem. But the most important missed point was correlations. As pointed out by Datta [4, chapter III], there has been much effort in order to find a suitable SCF that considers correlations. For example, to modify (66) to consider occupancies probabilities. As discussed in the introduction, Kang [16] and Meir and Wingreen [33] find a solution for the QD Green function that contains this type of correlation. In other words, one could go to scheme where a more accurate Green function for the QD is used together with a multielectron picture and corresponding master equation. These would mean to use the Anderson model with a Coulomb interaction *U* that is obtained from a SCF. We want to check if this point of view is correct. Work in this direction is in progress.

## Figures and Tables

**Figure 15:**
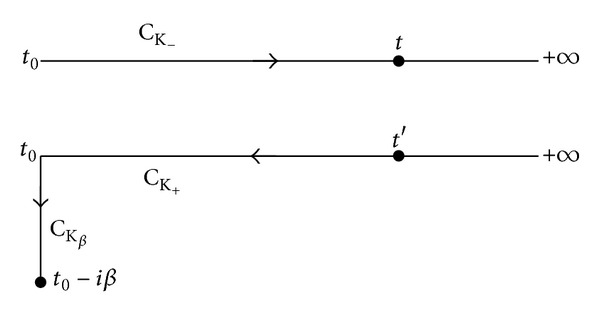
The contour C_K_ = C_K_−__ ∪ C_K_+__ runs on the real axis, but for clarity its two branches C_K_−__ and C_K_+__ are shown slightly away from the real axis. The contour C_K_ runs from *t*_0_ and returns to *t*_0_.

## Appendices

### A. Equation of Motion for the Keldysh-Green Function

In general, any Green-Keldysh function of two operators *A*(*t*) and *B*(*t*) function is given by

(A.1) $$\begin{array}{c} {\text{G}}_{A,B}\left(t,t^{\prime }\right)=-\text{i}\langle {\text{T}}_{\text{K}}A\left(t\right)B\left(t^{\prime }\right)\rangle , \end{array}$$

where the operator T_K_ acts on the Keldysh contour shown in Figure 15. A Heaviside function on the Keldysh contour is given by

(A.2) $$\begin{array}{c} \Theta \left(t,t^{\prime }\right)\equiv \{\begin{array}{cc} \Theta \left(t-t^{\prime }\right), & t\in {\text{C}}_{{\text{K}}_{-}},\;\;t^{\prime }\in {\text{C}}_{{\text{K}}_{-}};\\ 0, & t\in {\text{C}}_{{\text{K}}_{-}},\;\;t^{\prime }\in {\text{C}}_{{\text{K}}_{+}};\\ 1, & t\in {\text{C}}_{{\text{K}}_{+}},\;\;t^{\prime }\in {\text{C}}_{{\text{K}}_{-}};\\ \Theta \left(t^{\prime }-t\right), & t\in {\text{C}}_{{\text{K}}_{+}},\;\;t^{\prime }\in {\text{C}}_{{\text{K}}_{+}}, \end{array} \end{array}$$

whereas be derivative of the Heaviside function on the Keldysh contour is given by

(A.3) δ(t,t′)≡∂Θ(t,t′)∂t=−∂Θ(t,t′)∂t′={δ(t−t′),t∈CK−,  t′∈CK−;0,t∈CK−,  t′∈CK+;0,t∈CK+,  t′∈CK−;−δ(t−t′),t∈CK+,  t′∈CK+.

In general a function F(*t*, *t*) defined on the Keldysh contour is given by

(A.4) $$\begin{array}{c} \text{F}\left(t,t^{\prime }\right)\equiv \Theta \left(t,t^{\prime }\right){\text{F}}^{>}\left(t,t^{\prime }\right)+\Theta \left(t^{\prime },t\right){\text{F}}^{<}\left(t,t^{\prime }\right), \end{array}$$

where F^>^(*t*, *t*′) is the so-called greater component (greater Keldysh-Green function) and F^<^(*t*, *t*′) is the lesser component (lesser Keldysh-Green function) of F(*t*, *t*′).

Directly calculations can be carried out, in this way by deriving *t* or *t*′ and using Heisenberg equation of motion for the time evolution of the operators, *A*(*t*) = exp⁡(i*Ht*)*A*exp⁡(−i*Ht*), as

(A.5) $$\begin{array}{c} \frac{\partial A\left(t\right)}{\partial t}=-\text{i}\left[A\left(t\right),H\right], \end{array}$$

and one obtains

(A.6) i∂G(t,t′)∂t =δ(t,t′)〈[A(t),B(t)]∓〉−i〈TK[A(t),H]B(t′)〉,−i∂G(t,t′)∂t =δ(t,t′)〈[A(t),B(t)]∓〉+i〈TKA(t)[B(t′),H]〉.

If there is not an applied potential, that is, if *V*_*s*_ = *V*_*d*_ = 0, (see Figure 2), the whole system is at equilibrium, and one can use the usual commutator Green functions or equivalently the Matsubara or Temperature Green function to quantify correlations functions. In that case, (A.1) depends only on the time difference (*t* − *t*′). However, for times *t* > *t*_0_, once the potential difference has been applied, the simple dependence on the time differences no longer holds which signalizes a nonequilibrium situation. In that case, the Keldysh method applies.

### B. Equivalent Integral Equation on the Keldysh Countour on Figure 15

We encounter two cases (see (26) and (42)) where the general strategy to find an integral expression for the Keldysh-Green functions is the following: (1) one first considers the equation of motion for for the Keldysh-Green function of each system (two leads and the QD) (see (25) and (38)). (2) The resulting equation of motion in each case has a delta function as inhomogeneity. (3) The systems are connected in at *t*_0_, we have as a result two coupled equations of motion (see (20) and (41)). (4) These equations are converted into an equivalent integral equation on the Keldysh contour. (5) Straightforwardly, derivation of the integral equations results in the differential equation of motion.

### C. Calculation of Convolutions

In this appendix, we explain how we evaluate the some important convolutions used to calculate lesser Keldysh-Green functions [48, 49]. In general, a function given as a convolution on the Keldysh contour poses the definition given in Appendix A. One encounters situations where a Keldysh-Green function is given by a convolution of two other functions:

(C.1) $$\begin{array}{c} \text{P}\left(t,t^{\prime }\right)=\int_{{\text{C}}_{\text{K}}}{\text{d}}^{\prime \prime }\text{F}\left(t,t^{\prime \prime }\right)\text{G}\left(t^{\prime \prime },t^{\prime }\right). \end{array}$$

However, for evaluation of quantities like, for example, the current, one needs

(C.2) $$\begin{array}{c} {\text{P}}^{<}\left(t,t^{\prime }\right)=\int_{{\text{C}}_{\text{K}}}{{\text{d}}^{\prime \prime }\text{F}\left(t,t^{\prime \prime }\right)\text{G}\left(t^{\prime \prime },t^{\prime }\right)|}_{t{<}_{{\text{C}}_{\text{K}}}t^{\prime }}. \end{array}$$

One sees that the relative position of *t* and *t*′ divides the contour in three regions of integration: (1) *t*′′<_C_K__ *t*, (2) *t*<_C_K__ *t*′′ < *t*′, and (3) *t*′′>_C_K__ *t*′. This traduces into the following integrals:

(C.3) P<(t,t′)=∫t0tdt′′F>(t,t′′)G<(t′′,t′)|t′′<CKt +∫tt′dt′′F<(t,t′′)G<(t′′,t′)|t<CKt′′<t′ +∫t′t0dt′′F<(t,t′′)G>(t′′,t′)|t′′>CKt′.

With bookkeeping manipulations of the second integral, one obtains

(C.4) P<(t,t′) =∫t0t1dt′′[F(r)(t,t′′)G<(t′′,t′)+F<(t,t′′)G(a)(t′′,t′)],

where the retarded Keldysh-Green function F^(r)^(*t*, *t*′′) and the advance Keldysh-Green function G^(a)^(*t*, *t*′′) are given by

(C.5) $$\begin{array}{c} {\text{F}}^{\left(\text{r}\right)}\left(t,t^{\prime \prime }\right)=\Theta \left(t-t^{\prime \prime }\right)\left[{\text{F}}^{>}\left(t,t^{\prime \prime }\right)-{\text{F}}^{<}\left(t,t^{\prime \prime }\right)\right],\\ {\text{G}}^{\left(\text{a}\right)}\left(t^{\prime \prime },t^{\prime }\right)=-\Theta \left(t^{\prime }-t^{\prime \prime }\right)\left[{\text{G}}^{>}\left(t^{\prime \prime },t^{\prime }\right)-{\text{G}}^{<}\left(t^{\prime \prime },t^{\prime }\right)\right]. \end{array}$$

In the said way of reasoning one obtains

(C.6) P<{>}(t,t′)=∫−∞∞dt′′[F(r)(t,t′′)G<{>}(t′′,t′)       +F<{>}(t,t′′)G(a)(t′′,t′)].

With the definitions of a retarded/advanced Keldysh-Green function one easily obtains for P^(r)^(*t*, *t*′)

(C.7) P(r)(t,t′) =Θ(t−t′)∫−∞∞dt′′F(r)(t,t′′)[G>(t′′−t′)−G<(t′′,t′)]  +Θ(t′−t)∫−∞∞dt′′[F>(t−t′′)−F<(t,t′′)]G(r)(t′′,t′),

and therefore

(C.8) $$\begin{array}{c} {\text{P}}^{\left(\text{r}\right)}\left(t,t^{\prime }\right)=\overset{\infty }{\underset{-\infty }{\int }}\text{d}t^{\prime \prime }{\text{F}}^{\left(\text{r}\right)}\left(t,t^{\prime \prime }\right){\text{G}}^{\left(\text{r}\right)}\left(t^{\prime \prime },t^{\prime }\right). \end{array}$$

Similarly for P^(a)^(*t*, *t*′) one obtains

(C.9) $$\begin{array}{c} {\text{P}}^{\left(\text{a}\right)}\left(t,t^{\prime }\right)=\overset{\infty }{\underset{-\infty }{\int }}\text{d}t^{\prime \prime }{\text{F}}^{\left(\text{a}\right)}\left(t,t^{\prime \prime }\right){\text{G}}^{\left(\text{a}\right)}\left(t^{\prime \prime },t^{\prime }\right). \end{array}$$

### D. Calculation of Unperturbed Green Functions and Retarded Self-Energy

Below, we proceed to evaluate the unperturbed Green functions ${\text{g}}_{\eta \vec{k}\sigma }$ and ${\text{f}}_{\eta \vec{k}\sigma }$. To achieve this, it is necessary to introduce the chemical potential shift in each superconductor,

(D.1) $$\begin{array}{c} {ℋ}_{\eta }=H_{\eta }-\mu_{\eta }N_{\eta }, \end{array}$$

so that

(D.2) aηk→σ(t)≡eiHηtaηk→σe−iHηt=eiℋηt(eiμηtaηk→σe−iμηt)e−iℋηt=e−iμηt(eiℋηtaηk→σe−iℋηt)⟶e−iμηtaηk→σ(t),

due to

(D.3) $$\begin{array}{c} {\text{e}}^{\text{i}{ℋ}_{\eta }t}a_{\eta \vec{k}\sigma }{\text{e}}^{-\text{i}{ℋ}_{\eta }t}=a_{\eta \vec{k}\sigma }{\text{e}}^{-\text{i}{\text{E}}_{\eta \vec{k}}t},\\ {\text{e}}^{\text{i}N_{\eta }t}a_{\eta \vec{k}\sigma }{\text{e}}^{-\text{i}N_{\eta }t}=a_{\eta \vec{k}\sigma }{\text{e}}^{-\text{i}\mu_{\eta \vec{k}}t}, \end{array}$$

because [*N*_*η*_, *H*_*η*_] = 0. Consequently, the first unperturbed retarded Green function ${\text{g}}_{\eta \vec{k}\sigma }^{\left(\text{r}\right)}\left(t,t^{\prime }\right)$ is given by

(D.4) gηk→σ(r)(t,t′) =−iΘ(t−t′)〈{e−iμηtaηk→σ(t),eiμηt′aηk→σ†(t′)}〉 =−iΘ(t−t′)e−iμη(t−t′)  ×〈{uηk→γηk→σ(t)+σvηk→γηk→σ†(t),uηk→γηk→σ†(t′)     +σvηk→γη−k→−σ(t′)}〉 =−iΘ(t−t′)e−iμη(t−t′)[uηk→2e−iEηk→(t−t′)+vηk→2eiEηk→(t−t′)], =−iΘ(t−t′)[uηk→2e−i(Eηk→+μη)(t−t′)+vηk→2ei(Eηk→−μη)(t−t′)],

where the fermion operators $\gamma_{\eta \vec{k}\sigma }^{†}$, $\gamma_{\eta \vec{k}\sigma }$ create and annihilate the “Bogoliubov quasi-particles” and

(D.5) $$\begin{array}{c} \sigma =\{\begin{array}{cc} ↑ & =1\\ ↓ & =-1 \end{array} \end{array}$$

the spin index. They will be linear combinations of the creation and annihilation operators of the real electrons:

(D.6) $$\begin{array}{c} a_{\eta \vec{k}\sigma }\left(t\right)=u_{\eta \vec{k}}\gamma_{\eta \vec{k}\sigma }\left(t\right)+\sigma v_{\eta \vec{k}}\gamma_{\eta \vec{k}\sigma }^{†}\left(t\right),\\ a_{\eta \vec{k}\sigma }^{†}\left(t^{\prime }\right)=u_{\eta \vec{k}}\gamma_{\eta \vec{k}\sigma }^{†}\left(t^{\prime }\right)+\sigma v_{\eta \vec{k}}\gamma_{\eta -\vec{k}-\sigma }\left(t^{\prime }\right). \end{array}$$

Applying the Fourier transformations to ${\text{g}}_{\eta \vec{k}\sigma }^{\left(\text{r}\right)}\left(t,t^{\prime }\right)$

(D.7) $$\begin{array}{c} {\text{g}}_{\eta \vec{k}}^{\left(\text{r}\right)}\left(t,t^{\prime }\right)=\overset{\infty }{\underset{-\infty }{\int }}\frac{\text{d}\omega }{2\pi }{\text{e}}^{-\text{i}\omega (t-t^{\prime })}{\text{g}}_{\eta \vec{k}}^{\left(\text{r}\right)}\left(\omega \right), \end{array}$$

with

(D.8) $$\begin{array}{c} {\text{g}}_{\eta \vec{k}}^{\left(\text{r}\right)}\left(\omega \right)=\frac{u_{\eta \vec{k}}^{2}}{\omega -{\text{E}}_{\eta \vec{k}}-\mu_{\eta }+\text{i}0^{+}}+\frac{v_{\eta \vec{k}}^{2}}{\omega +{\text{E}}_{\eta \vec{k}}-\mu_{\eta }+\text{i}0^{+}}. \end{array}$$

Therefore we have

(D.9) Vη2∑k→gηk→(r)(ω) =12Vη2∑k→(1ω−Eηk→−μη+i0++1ω+Eηk→−μη+i0+) =Vη2P∑k→ω−μη(ω−μη)2−Eηk→2  −12iπVη2∑k→[δ(ω−μη−Eηk→)+δ(ω−μη+Eηk→)] =−Nη(0)Vη2P∫−∞∞dξω−μηξ2+Δ2−(ω−μ)2  −12iπVη2∑k→δ(|ω−μη|−Eηk→).

Finally,

(D.10) Σ(r)(ω)≡Vη2∑k→gηk→(r)(ω)=−Γη[ω−μηΔηζ(Δη,ω−μη)               +iζ(ω−μη,Δη)],

where

(D.11) $$\begin{array}{c} \Gamma_{\eta }=\pi N_{\eta }\left(\omega -\mu_{\eta }\right)V_{\eta }^{2}\approx \pi N_{\eta }\left(0\right)V_{\eta }^{2},\\ \zeta \left(\omega ,\omega^{\prime }\right)\equiv \Theta \left(\left|\omega |-|\omega^{\prime }\right|\right)\frac{\left|\omega \right|}{\sqrt{\omega^{2}-\omega^{\prime 2}}}. \end{array}$$

Γ_*η*_ = *πN*_*η*_(*ω* − *μ*_*η*_)*V*_*η*_^2^ ≈ *πN*_*η*_(0)*V*_*η*_^2^ are the coupling constants between the leads and the quantum dot in the wide band limit (WBL). *N*_*η*_(0) is the density of states at the *η* Fermi level and f(*ω*) is the Fermi-Dirac distribution function.

The WBL means that the width of the electronic energy bands of the leads is the largest energy. The density of states in the contacts varies on a scale of Fermi energy. These scales are of order 1–10 eV (∼10^4^-10^5^ K) which are much larger than the energies involved in the quantum dot ∼meV ~ 10 K. Furthermore, Γ_*η*_(*ω* − *μ*_*η*_) varies slowly with *ω* − *μ*_*η*_ and the prefactor *𝒟*_*η*_(*ω* − *μ*_*η*_) varies in the range of wide band and changes *ω* − *μ*_*η*_ on the average of $|V_{\eta \vec{k}}{|}^{2}$ occur on the order of meV. Therefore, we ignore the dependence of *ω* − *μ*_*η*_ in Γ_*η*_(*ω* − *μ*_*η*_). The WBL establishes that an electron in the dot decays in an continuum of states of the leads and is sufficient condition for the existence of a stationary state, as has been shown rigorously in [50].

### E. Calculation of the Lesser Green Function and Lesser Self-Energy

Consider

(E.1) gηk→σ<(t,t′) =i〈{eiμηt′aηk→σ†(t′),e−iμηtaηk→σ(t)}〉 =ieiμη(t−t′)〈[uηk→γηk→σ†(t′)+σvηk→γη−k→−σ(t′)]        ×[uηk→γηk→σ(t)+σvηk→γη−k→−σ†(t)]〉 =i[uηk→2e−i(Eηk→+μη)(t−t′)f(Eηk→)    +vηk→2ei(Eηk→−μη)(t−t′)f(−Eηk→)].

Applying the Fourier transformations,

(E.2) $$\begin{array}{c} {\text{g}}_{\eta \vec{k}\sigma }^{<}\left(t,t^{\prime }\right)=\overset{\infty }{\underset{-\infty }{\int }}\frac{\text{d}\omega }{2\pi }{\text{e}}^{-\text{i}\omega (t-t^{\prime })}{\text{g}}_{\eta \vec{k}}^{<}\left(\omega \right), \end{array}$$

with

(E.3) gηk→σ<(ω)=2πi[uηk→2δ(ω−Eηk→−μη)    +vηk→2δ(ω+Eηk→−μη)]f(ω−μη).

Therefore we have

(E.4) Vη2∑k→gηk→σ<(ω) =2πiVη2∑k→[uηk→2δ(ω−Eηk→−μη)        +vηk→2δ(ω+Eηk→−μη)]×f(ω−μη) =2πiVη212∑k→δ(|ω−μη|−Eηk→)f(ω−μη).

Finally,

(E.5) $$\begin{array}{c} \Sigma^{<}\left(\omega \right)\equiv V_{\eta }^{2}\underset{\vec{k}}{\sum }{\text{g}}_{\eta \vec{k}}^{<}\left(\omega \right)=2\text{i}\Gamma_{\eta }\zeta \left(\omega -\mu_{\eta },\Delta_{\eta }\right)\text{f}\left(\omega -\mu_{\eta }\right). \end{array}$$

### F. Calculation of the Retarded (Tilde) Self-Energy

Consider

(F.1) f~ηk→σ(r)(t,t′) =−iΘ(t−t′)〈{eiμηtaηk→σ(t),eiμηt′aη−k→−σ†(t′)}〉 =−iΘ(t+t′)eiμη(t+t′)  ×〈{uηk→γηk→σ†(t)+σvηk→γηk→σ†(t),uηk→γη−k→−σ(t′)    +σvηk→γηk→σ†(t′)}〉 =−iΘ(t−t′)e−2iμηte−iμη(t−t′)σvηk→uηk→  ×[eiEηk→(t−t′)+eiEηk→(t−t′)] =−iΘ(t−t′)σΔη2Eηk→  ×[e−i(Eηk→−μη)(t−t′)+ei(Eηk→+μη)(t−t′)].

Applying the Fourier transformations

(F.2) $$\begin{array}{c} V_{\eta }^{2}\underset{\vec{k}}{\sum }{\tilde{\text{f}}}_{\eta \vec{k}\sigma }^{\left(\text{r}\right)}\left(t,t^{\prime }\right)=\overset{\infty }{\underset{-\infty }{\int }}{\text{e}}^{-2\text{i}\mu_{\eta }t}\sigma \frac{\text{d}\omega }{2\pi }{\text{e}}^{-\text{i}\omega (t-t^{\prime })}{\tilde{\Xi }}_{\eta \vec{k}}^{\left(\text{r}\right)}\left(\omega \right), \end{array}$$

with

(F.3) Vη2∑k→Ξ~ηk→(r)(ω) =Δη2Eηk→[1ω−Eηk→−μη+i0++1ω+Eηk→−μη+i0+] =Vη2∑k→Δη2Eηk→[1ω−Eηk→−μη+i0++1ω+Eηk→−μη+i0+] =Vη2Δη∑k→[P1ξηk→2+Δη2−(ω−μη)2+iπ2(ω−μη)       ×δ(|ω+μη|−Eηk→)] =Nη(0)Vη2Δη×P∫−∞∞dξ1ξ2+Δ2−(ω+μ)2  +iπVη22(ω+μη)∑k→δ(|ω+μη|−Eηk→).

Finally,

(F.4) $$\begin{array}{c} {\tilde{\Xi }}_{\eta \vec{k}}^{\left(\text{r}\right)}\left(\omega \right)=\Gamma_{\eta }\left[\zeta \left(\Delta_{\eta },\omega +\mu_{\eta }\right)+\text{i}\frac{\Delta_{\eta }}{\omega +\mu_{\eta }}\zeta \left(\omega +\mu_{\eta },\Delta_{\eta }\right)\right]. \end{array}$$

### G. Calculation of the Lesser (Tilde) Self-Energy

Consider

(G.1) f~ηk→σ<(t,t′) =i〈{e−iμηt′aη−k→−σ(t′),e−iμηtaηk→σ(t)}〉 =ie−iμη(t+t′)〈[uηk→γη−k→−σ(t′)−σvηk→γη−k→−σ†(t′)]          ×[uηk→γηk→σ(t)+σvηk→γη−k→−σ†(t)]〉 =ie−2iμηteiμη(t−t′)[σuηk→vηk→ei(Eηk→)(t−t′)f(−Eηk→)           +σvηk→uηk→e−i(Eηk→)(t−t′)f(Eηk→)] =ie−2iμηtσΔη2Eηk→[ei(Eηk→+μη)(t−t′)f(−Eηk→)           +e−i(Eηk→−μη)(t−t′)f(Eηk→)].

Therefore we have

(G.2) $$\begin{array}{c} V_{\eta }^{2}\underset{\vec{k}}{\sum }{\tilde{\text{f}}}_{\eta \vec{k}\sigma }^{<}\left(t,t^{\prime }\right)=\overset{\infty }{\underset{-\infty }{\int }}{\text{e}}^{-2\text{i}\mu_{\eta }t}\sigma \frac{\text{d}\omega }{2\pi }{\text{e}}^{-\text{i}\omega (t-t^{\prime })}{\tilde{\Xi }}_{\eta \vec{k}}^{<}\left(\omega \right), \end{array}$$

with

(G.3) Ξ~ηk→<(ω) =Vη2∑k→πiEηk→[δ(ω+Eηk→+μη)f(−Eηk→)         −δ(ω−Eηk→+μη)f(Eηk→)] =Vη2∑k→πiEηk→[δ(ω+Eηk→+μη)          −δ(ω−Eηk→+μη)]f(ω+μη) =−2iΔηω−μη[πVη22∑ηδ(|ω+μη|−Eηk→)]f(ω+μη).

Finally,

(G.4) $$\begin{array}{c} {\tilde{\Xi }}_{\eta \vec{k}}^{<}\left(\omega \right)=-2\text{i}\Gamma_{\eta }\frac{\Delta_{\eta }}{\omega +\mu_{\eta }}\zeta \left(\omega +\mu_{\eta },\Delta_{\eta }\right)\text{f}\left(\omega +\mu_{\eta }\right). \end{array}$$
